# An Intervention to Improve Mental and Physical Health of Undergraduate Nursing Students

**DOI:** 10.1177/08445621241248308

**Published:** 2024-05-05

**Authors:** Sylwia Ciezar Andersen, Tavis Campbell, Deborah White, Kathryn King-Shier

**Affiliations:** 1 Faculty of Nursing, 2129 University of Calgary, Calgary, Canada; 2 Department of Psychology, 2129 University of Calgary, Calgary, Canada; 3 Faculty of Nursing, Qatar University, Doha, Qatar

**Keywords:** Nursing student, web-based, undergraduate, depression, anxiety, stress

## Abstract

**Background:**

Nursing students experience poorer mental and physical health relative to students in other health-related disciplines and young adults of similar age outside post-secondary school. Compromised mental and physical health has numerous negative impacts on nursing students and can result in burnout and development of chronic diseases.

**Purpose:**

To determine whether an asynchronous online yoga intervention would improve mental and physical health of students.

**Methods:**

An asynchronous online 6-week yoga intervention was carried out between January and December 2021, using a pre/post design. Participants’ symptoms of depression, anxiety, stress, and self-compassion were assessed using the Depression, Anxiety, and Stress Scale and Self-Compassion Scale and core endurance was assessed using the Mackenzie Core Endurance Test prior to commencement and at the conclusion of the program.

**Results:**

Of 114 participants, 68 completed the online program and pre and post measures showed that the mean depression, anxiety, stress, self-compassion and core endurance scores improved significantly (*p*<0.001) between baseline and study completion.

**Conclusion:**

A six-week virtual yoga program significantly improved mental and physical health of undergraduate nursing students. Targeted modifications to the yoga program might enhance participant retention.

## 1. Introduction

Mental and physical health are inter-related components of overall health (CDC, 2022; Taylor et al., 1985). Nurses have poorer mental and physical health (Blake & Chambers, 2012; Deasy et al., 2016) relative to the general public (Blake & Chambers, 2012) and other professionals (Kumbrija et al., 2007) exacerbating already difficult recruitment and retention of nurses globally. Symptoms of depression, anxiety, or stress that are in excess of population norms are indicative of poor mental health (Mahmoud et al., 2012). Sedentary behavior is a proxy for poor physical health, poor core endurance, and musculoskeletal injury (Chen et al., 2009; Lurati, 2018). Clinical depression in nurses in the United States (US) is more than double (25%; Olaya et al., 2021) than that of the national average (9%; National Institute of Mental Health, 2023). Similarly, 36% of Canadian nurses have depressive disorders, which is more than four times the national average (7.6%; Statistics Canada, 2023; Stelnicki & Carleton, 2021). The findings of a recent systematic review and meta-analysis revealed a 35% pooled prevalence of depressive symptoms in nurses worldwide (Al Maqbali et al., 2021). Up to three times higher than the national averages (Cheung & Yip, 2015), the prevalence of symptoms of anxiety (39–42%) and stress (41–50%) in nurses worldwide is equally alarming (Al Maqbali et al., 2021; Maharaj et al., 2019). There is also a high prevalence of inactivity/sedentary behavior in up to 76.5% of nurses (Letvak, 2013).

Nurses’ poor mental and physical health may begin as early as when they are undertaking undergraduate education (Bartlett et al., 2016; Hosseini et al., 2013; Meyer & Shatto, 2018; Thomas, 2022; Wills & Kelly, 2017). Nursing students experience poorer mental and physical health relative to students in other health-related disciplines and young adults of similar age outside post-secondary school (Jimenez et al., 2010; McDermott et al., 2021; Morgan & Graff, 2019; Thomas, 2022). Differences in stress scores between nursing students and their non-nursing counterparts have been reported. A survey of 2326 undergraduate students revealed 57% of nursing students versus 49% of non-nursing students (*p*=0.001) rated their stress as ‘severe’ on the Student Stress Inventory (Thomas, 2022). The National College Health Assessment revealed that 17.6% of nursing students reported symptoms of ‘tremendous stress’ compared to 6.9% of non-nursing students (*p*<0.001) and nursing students had significantly more anxiety diagnoses and treatment for symptoms of anxiety relative to the general student body (*p*=0.006; National College Health Assessment, 2019). Similar trends were seen in a survey of Canadian nursing students (*n*=437) who had significantly higher depression, anxiety, and stress scores (*p*<0.005) than their non-nursing (*n*=1870) counterparts (Chernomas & Shapiro, 2013). Unmanaged symptoms of depression, anxiety, or stress have a variety of negative impacts on the individual nursing student which mayinclude burnout and development of chronic diseases (Chernomas & Shapiro, 2013; Jimenez et al., 2010; McDermott et al., 2021; Morgan & Graff, 2019; Thomas, 2022; WHO, 2021).

Poor mental health is associated with poor physical health, and sedentary behavior specifically (WHO, 2021). Nursing students report lower levels of physical activity than students in other health professions. A survey of 361 nursing and medical students in the United Kingdom revealed that 48% of student nurses reported being sedentary compared to 38% of medical students (Blake et al., 2017), and nursing students were significantly less physically active than paramedic students (*p*=0.009; Micalos et al., 2017). Up to 50% of nursing students are sedentary (Blake et al., 2017; Deasy et al., 2016; Irazusta et al., 2006; Lehmann et al., 2014), over 50% report having no established exercise routine, and 37.8% are above their ideal Body Mass Index (BMI; Pugh et al., 2019). Despite the upsurge of ergonomic lift/transfer technology and no-lift policies (Choi & Brings, 2016), forty percent of new graduate nurses report sprains, strains, and back injuries within two years of commencing practice (Brewer et al., 2012; Craft et al., 2017), likely related to their sedentary behavior and poor core endurance. Over the last 20 years, studies have linked poor core endurance with musculoskeletal pain and injury, particularly in the lower back (Carpes et al., 2008; Cholewicki et al., 2005; D’hooge et al., 2013; Hodges & Richardson, 1998; Hungerford et al., 2003; Huxel Bliven & & Anderson, 2013).

### 1.1 Background

The practice of yoga has been examined as a mechanism to improve symptoms of depression, anxiety and stress (Buffart et al., 2012; Cramer et al., 2013; Field, 2016; Pascoe & Bauer, 2015; Riley & Park, 2015; Sharma, 2014; Sharma & Haider, 2013) and to improve physical health (Field, 2016; Govindaraj et al., 2016; Raub, 2002; Ross & Thomas, 2010; Sengupta, 2012). While many different styles of yoga exist, yoga practice generally consists of three key aspects: physical postures (asanas), breathing work (pranayama), and mindfulness of movement. Yoga practice has improved mental and physical health in a variety of groups including healthcare professionals and students (Chong et al., 2011; Ciezar-Andersen et al., 2021; Field, 2016; Gaskins et al., 2014; Pascoe et al., 2017; Pascoe & Bauer, 2015; Riley & Park, 2015; Yi et al., 2021). Engaging in yoga improved symptoms of depression (*p*<0.001), anxiety (*p*<0.001), and stress (*p*<0.001) in a sample of breast cancer patients (Banerjee et al., 2007). Similarly, yoga activity improved symptoms of anxiety, depression, social dysfunction, and somatic symptoms (*p*<0.001) in a sample of medical students (Bansal et al., 2013). Yoga also improved self-compassion and symptoms of stress (*p*<0.05) in healthcare students (Beck & Verticchio, 2014), and increased stress resilience and commitment to self-care practices in nursing students (Clark, 2018). A significant reduction in musculoskeletal pain and disability and improvement spinal flexibility (*p*<0.01), indicators of physical health, were demonstrated in nurses who practiced yoga (Patil et al., 2018).

Studies examining the effects of an in-person yoga intervention on healthcare professionals and students including dental interns (Deolia et al., 2017; Shankarapillai et al., 2012), nursing students (Craighead, 2015; Kim, 2014; Mathad et al., 2017), medical students (Gopal et al., 2011; Malathi et al., 1998; Malathi & Damodaran, 1999; Parshad et al., 2011; Prasad et al., 2016; Simard & Henry, 2009), and dental hygiene students (Monson et al., 2017), revealed similar trends of improved symptoms of mental and physical health. However, based on a recent systematic review (Ciezar-Andersen et al., 2021), whether an online yoga intervention can improve nursing students’ symptoms of depression, anxiety, stress, and self-compassion has not been previously explored. Symptoms of stress alone were explored in two studies (Clark, 2018; Kim, 2014), and stress and self-compassion were explored in one study (Mathad et al., 2017) offering in-person interventions. All reviewed studies had methodological issues including a lack of reporting intervention details to ensure reproducibility (Ciezar-Andersen et al., 2021). Thus, the purpose of this study was to determine whether a tailored online yoga program would improve mental and physical health parameters of undergraduate nursing students. The investigators hypothesized that there would be improvement in symptoms of depression, anxiety, stress, self-compassion (a potential mechanism through which yoga reduces perceived stress; Kinchen et al., 2020; Mathad et al., 2017; Riley & Park, 2015), and core endurance (linked to lowering the risk of musculoskeletal injury; Akuthota & Nadler, 2004; McGill, 2015; Willson et al., 2005), following the intervention.

## 2. Methods

### 2.1 Aims

The aim of this study aimed to determine whether a tailored on-line yoga program would improve mental (i.e., symptoms of depression, anxiety, and stress as well as self-compassion) and physical (i.e., core endurance) health of undergraduate nursing students.

### 2.2 Design and intervention

This was a single group pre-post study design to assess the effects of the yoga intervention on participants. The primary investigator (SCA), a certified yoga teacher (RYT-200) with over 20 years of yoga experience designed a 6-week program of pre-recorded yoga classes. Classes were selected to target participants with beginner level of experience (with attention to alignment) and progressed to more intermediate poses after the first two weeks. The Template for Intervention Description and Replication (TIDieR) Checklist (Hoffmann et al., 2016) was used to guide the design of the intervention and report findings (Table 1).

**Table 1.** TIDieR checklist.

TIDierR Criterion	Description
Brief Name	Online yoga intervention for undergraduate nursing students.
Why	Nursing students have poorer mental and physical health than their counterparts. Yoga has been shown to improve mental and physical health. Participant depression, anxiety, stress, and core endurance measured pre and post intervention.
What	Pre-recorded online yoga sessions.
Who Provided	RYT-200 certified yoga instructors with minimum 3 years teaching experience.
How	Delivered as a pre-recorded online class to individual participants.
Where	In-home. Participants provide own yoga mat and internet access, including audio.
When and How Much	**Week 1 Module:** Beginner Yoga Basics Class 1: Introduction to Yoga/Foundational Concepts/ Foundational Poses 1 (67 min) Class 2: Foundational Poses 2/Sun Salutations (58 min) Class 3: Full Length Class (47 min) **Week 2 Module:** Basic Strength Class 1: Intro to Program/Basic Flexibility/Basic Conditioning (53 min) Class 2: Basic Strengthening Flow (38 min) Class 3: Repeat Basic Strengthening Flow (38 min) **Week 3 Module:** Yoga Strong Challenge Class 1: Core/Legs and Glutes (45 min) Class 2: Arms and Chest/Balance (40 min) Class 3: Spine/Full Body Flow/Mind (59 min) **Week 4 Module:** Intro to Vinyasa Class 1**:** Yoga Strong Challenge; Full Body Flow/Mind (41 min) Class 2**:** Intro to Vinyasa Yoga; Intro, Basics, Warrior Series, Expand your Foundation, Sun Salutation Flow (50 min) Class 3**:** Intro to Vinyasa Yoga; Full-Length Flow (39 min) **Week 5 Module:** Yoga Strong Challenge Class 1**:** Yoga Strong Challenge; Full Body Flow/Mind (41 min) Class 2: Stress Less (25 min) Class 3: Basic Strength; Basic Strengthening Flow (38 min) **Week 6 Module:** Strengthening Flow Class 1: Back to Presence (40 min) Class 2: Basic Strength; Basic Strengthening Flow (38 min) Class 3: Full Body Flow/Mind (41 min)
Tailoring	Participants complete 3 classes per week as scheduled with 1 day of rest between classes. 2 days of rest between modules.
Modifications	No modifications were required.
How Well	Participant fidelity was assessed via a log of date of completion of each class.

Mental and physical health were assessed before the program began and at the 6-week conclusion. The pre-recorded classes were accessible to participants online free of charge through yogiapproved.com. Following appropriate ethics approval, all undergraduate nursing students across six Alberta post-secondary institutions were invited to participate in the study. Length, intensity, flexibility, and content of classes were designed to appeal to novice participants with limited time (Table 1).

Classes were developed based on a combination of Hatha, Iyengar, and Vinyasa yoga and had a duration of 25 to 67 min (mode 45 min; Table 1). Participants were encouraged to contact the yoga instructor (SCA) with any alignment or positioning inquiries. Classes were low to moderate in intensity, progressing from beginner to more intermediate poses (held for 30–60 s), consisted of asana, breathwork, and meditation, with a mean class length of 40 min (Table 1). Participants were instructed to complete three classes per week, and to refrain from commencing other physical activitiesduring the 6-week duration of the program.

### 2.3 Sample/participants

Participants were recruited between January and November, 2021. Inclusion criteria were enrollment as a nursing student at one of the Alberta sites and having a computer with reliable internet access. Exclusion criteria were current participation in moderate or strenuous exercise (categorized by the Harvard School of Health, 2023) three or more times per week, any recent (within past six months) yoga practice, acute disease including upper respiratory illness, acute arthritis, acute bronchitis, cervical spondylosis, or extensive physical disability. Potential participants were screened for eligibility by SCA during initial contact. A priori sample size calculation using G*Power indicated 64 participants were required for a conservative effect size of 0.357 (based on effect size of stress reduction reported in a previous yoga intervention with young adults; Gard et al., 2012), with an alpha of 0.05 and power of 0.80. Assuming a 20% attrition rate, we aimed to have 77 participants.

### 2.4 Data collection

Demographic characteristics (gender, age, year of program, institution; Table 2) of the study sample were collected electronically via QualtricsXM software [2022; Qualtrics; Provo, UT, USA], using an investigator developed tool. Participants were anonymized and assigned a unique numerical identifier to link data to responses during analysis. The TIDieR Checklist (Hoffmann et al., 2016) was used to guide data reporting (Table 1). This includes reporting of a clear ‘dose’ and providing sufficient information to ensure study reproducibility; characteristics lacking in previous studies with students in the health professions (Ciezar-Andersen et al., 2021).

**Table 2.** Demographic characteristics (*n*=68).

Characteristic	N (%)
**Sex**	
Female	64 (94%)
**Age (years)**	
17–20	25 (37%)
21–25	25 (37%)
26–30	7 (10%)
31–35	4 (6%)
36 and over	7 (10%)
**Institution**	
1	35 (52%)
2	17 (25%)
3	2 (3%)
4	4 (6%)
5	7 (10%)
6	3 (4%)
**Year Program**	
1	24 (35%)
2	17 (25%)
3	15 (22%)
4	12 (18%)

Pre-intervention baseline data were collected using the Depression, Anxiety, and Stress Scale (DASS-42; Lovibond & Lovibond, 1995), the Self-Compassion Scale (SCS; Neff, 2003), as well as the Mackenzie Core Endurance Test (MCET; Mackenzie, 2005) directly before the participant commenced the program and within 7 days of completing the 6-week program. Inter-rater reliability of the the pre and post MCET data was assured as the data were collected by the same assessor (SCA). Intra-rater reliability was assured by having an additional assessor timing 10% of all sessions at random, and observations were compared. To monitor adherence, participants kept a dated log of all classes completed which they turned in upon completion of the program.

The DASS-42 includes three self-report scales designed to measure the negative emotional states of depression, anxiety, and stress (DASS-42; Lovibond & Lovibond, 1995). Each of the three scales contains 14 items, which are divided into subscales of 2–5 items with similar content. Internal consistency coefficients of the original DASS-42 subscales were 0.83 for depression, 0.79 for anxiety, and 0.81 for stress in a sample of undergraduate students (Alansari, 2021).

The SCS is a 26-item self-report scale that represents the thoughts, emotions, and behaviors associated with self-compassion which are further divided into six subcategories, three positive (self-kindness, common humanity, mindfulness) and three negative (isolation, over-identification, and self-judgement) subcategories. Internal consistency coefficients of these components range between 0.75 and 0.81 (Neff, 2003).

Core endurance was measured using the MCET, a reliable predictor of core stability and physical health, that exhibits good construct and test-retest validity and reliability with an ICC of 0.97 (95% confidence interval: 0.94–0.99), and a smallest detectable difference of 5.85 s indicating sensitivity to 3% of actual change in core muscle endurance (Mackenzie, 2005; Tong et al., 2014). Participants met with the assessor live via Zoom. The timed test involved positioning of the participant in full side view with prompts to change position after 60 s (based on the MCET protocol; Mackenzie, 2005) while in full view of the assessor via camera.

### 2.5 Ethical considerations

The study was approved by the Conjoint Health Research Ethics Board (REB19–1640) at the University of Calgary and the relevant ethics boards at other participating institutions. A recruitment email with contact details was sent to all undergraduate nursing students by administrators of the participating Alberta post-secondary institutions. Following a face-to-face explanation of the program requirements, written consent via signature was obtained from agreeable volunteers. Participation was voluntary and participants could withdraw at any time.

### 2.6 Data analysis

Data were exported from Qualtrics to SPSS, Version 28 (IBM Corp., 2021) for analysis. Demographic characteristics of the study sample were examined using descriptive statistics and are reported as number/total (percentages) unless otherwise indicated (see Table 2). The raw scores for depression, anxiety, and stress were categorized into three groups (normal, mild-moderate, severe) and the scores for self-compassion and core endurance were similarly categorized (below average, average, and above average), based on scoring categorizations developed with North American post-secondary students (Alansari, 2021; Brigham & Chase, 2014; Neff, 2003). The differences between pre- and post-intervention mental (depression, anxiety, stress, self-compassion) and physical (core endurance) scores were examined using paired t-tests. Based on a two-tailed test, a p-value of less than 0.05 was considered statistically significant. Only participants with complete data sets were included in the analysis.

### 2.7 Reliability and validity

The instruments used in this study had previously demonstrated good reliability and validity, as reported above. In the current sample, the DASS-42 Cronbach's alpha for depression, anxiety, and stress was 0.947, 0.910, and 0.933 respectively. For the SCS subscales in the current sample, the Cronbach's alphas were as follows: for self-kindness; 0.815, self-judgement; 0.853, common humanity; 0.841, isolation; 0.740, mindfulness; 0.754 and over-identification; 0.751.

## 3. Results

### 3.1 Participant characteristics

**Figure 1:**
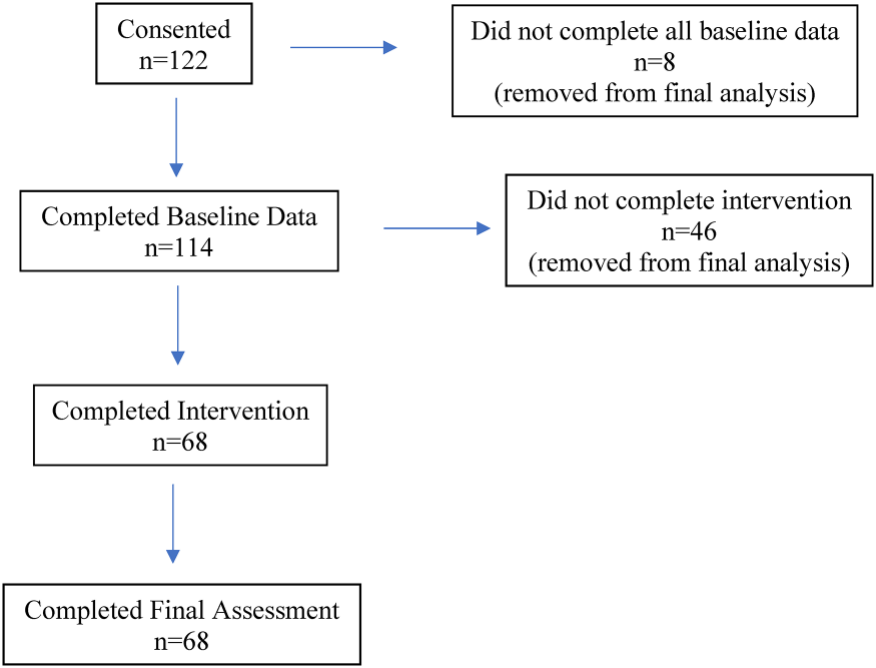
Participant recruitment and retention flow chart.

Of the 122 undergraduate nursing students who initially consented to participate in the study, 68 (56%) completed all assigned classes (as per schedule logs reviewed by SCA upon completion) and data collection points (Figure 1). The majority (61%) of participants who withdrew from the study did so at the three-week point. Of the 68 participants who completed the study, four were male. The majority of participants (74%) were between 17–25 years old, and 60% were enrolled in their first or second year of the nursing program at the time of the study (Table 2). There were no significant differences in demographic characteristics between participants who completed the study compared to those who withdrew.

### 3.2 Scores over time

**Figure 2:**
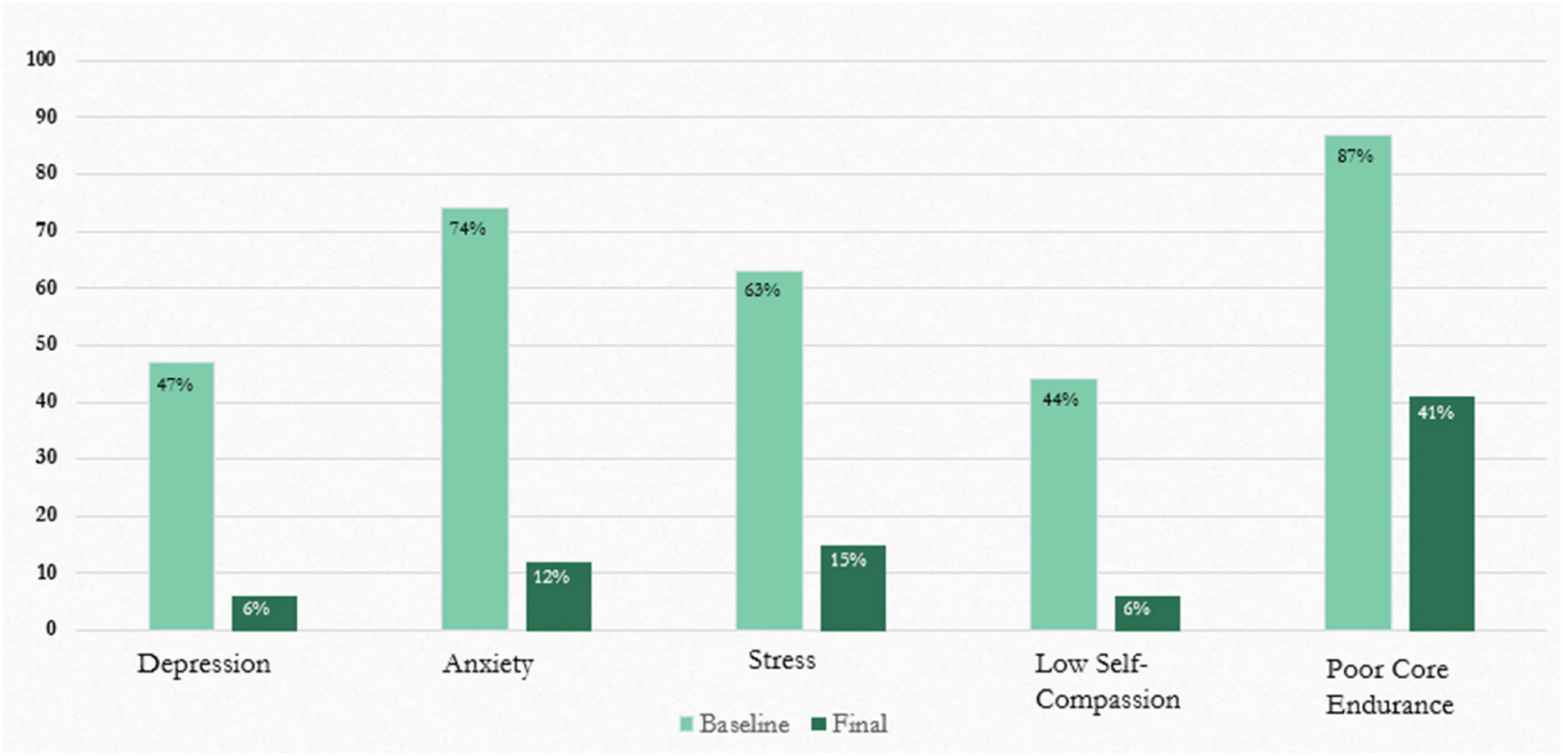
Percentage of participants outside normal range before and after intervention.

Statisitcal significance (*p*< 0.001) was attained for the differences in all scores over time. As seen in Table 3, the percentage of participants whose poor mental health scores fell above the normative range (Table 4) decreased following the intervention. High depression scores (47% at baseline to 6% following the intervention), high anxiety scores (74% to 12%), and high stress scores (63% to 15%; Figure 2, Table 4) decreased significantly following the intervention. Additionally, all improvements in scores had large effect sizes (>1; Table 4). A considerable proportion of participants reported symptoms of severe depression (19.1%), anxiety (27.9%), and stress (23.6%; note that the study protocol included appropriate referral for participants whose depression, anxiety or stress scores were in the severe range) at baseline. Forty four percent of participants had low self-compassion scores (below 3; Table 4) at baseline, but only 6% fell in this range following the intervention. Eighty seven percent of participants’ core endurance scores fell below normative values (Table 4) at baseline, which decreased to 41% following completion of the intervention. The mean depression, anxiety, stress, self-compassion and core endurance scores improved significantly (*p*<0.001) between baseline and study completion to within normative ranges (see Tables 3, 4).

**Table 3.** Depression, anxiety, stress, self-compassion, and core endurance score classification at baseline and post-intervention (*n*=68).

Variable	Baseline N (%)	Post-intervention N (%)
**Depression**		
Severe	13 (19.1%)	2 (3%)
Mild-Moderate	19 (27.9%)	2 (3%)
Normal	36 (52.9%)	64 (94.1%)
**Anxiety**		
Severe	19 (27.9%)	6 (8.9%)
Mild-Moderate	31 (45.6%)	9 (13.3%)
Normal	18 (26.5%)	53 (77.9%)
**Stress**		
Severe	16 (23.6%)	1 (1.5%)
Mild-Moderate	27 (39.7%)	9 (13.3%)
Normal	26 (36.8%)	58 (85.3%)
**Self-Compassion**		
Below Average	30 (44.1%)	4 (5.9%)
Average	31 (45.6%)	30 (44.1%)
Above Average	7 (10.3%)	34 (50%)
**Core Endurance**		
Below Average	59 (86.8%)	28 (41.2%)
Average	3 (4.4%)	9 (13.2%)
Above Average	6 (8.8%)	31 (45.6%)

**Table 4.** Normative and mean variable scores at baseline and post-intervention.

Variable	Possible Range	Normal Range	Baseline Mean (SD)	Final Mean (SD)	P Value	Effect Size (Cohen's δ)
Depression	0–28	0–9	11.90 (9.63)	4.03 (4.51)	<0.001	−1.11
Anxiety	0–20	0–7	11.84 (7.70)	5.31 (4.89)	<0.001	−1.04
Stress	0–34	0–14	18.74 (8.71)	9.09 (5.78)	<0.001	−1.34
Self- Compassion	1–5	3	2.75 (0.68)	3.57 (0.75)	<0.001	1.15
Endurance (sec)	0–180	96–116	68.19 (25.15)	108.51 (36.65)	<0.001	1.30

## 4. Discussion

The major findings from this study show that a yoga intervention has the potential to improve the mental and physical health of undergraduate nursing students. Improvements were identified in all mental health outcomes (symptoms of depression, anxiety, stress, self-compassion; DASS, SCS) as well as enhanced physical health (measured by core endurance; MCET) following the intervention. There is a strong link between increased core endurance and back injury prevention (D’hooge et al., 2013; Huxel Bliven & & Anderson, 2013; Rickman et al., 2012; Silfies et al., 2015; Smith et al., 2008). To the best of our knowledge, we are the first authors to report the effect size of statistically significant improvements in mental and physical health parameters in a group of nursing students. While a Randomized Controlled Trial (RCT) by Mathad et al. (2017) of once weekly yoga intervention for nursing students over a period of eight weeks demonstrated a significant improvement in self-compassion (*p*=0.037), and a 7% decrease in perceived stress scores of participants following the intervention, the difference in stress scores did not attain statistical significance (*p*=0.066). Riley et al. (2017) reported similar findings in a pre-post study following an eight-week (once weekly) yoga intervention for mental healthcare providers, with significantly decreased symptoms of depression (*p*=0.002) and stress (*p*=0.001), and increase in self-compassion (*p*=0.003). Similarly, following a seven-week yoga intervention, in a narrative inquiry (Clark, 2018) 82 student nurses reported enhanced well-being, self-care, and stress reduction. Contrary to our findings, in a sample of 73 nursing students, Kinchen et al. (2020) reported an increase in stress in both the control and yoga intervention groups, potentially due to the timing of their final assessment coinciding with exam times.

While core endurance following a yoga intervention has not been previously measured in nursing students, our findings are similar to those with other participant groups. In an RCT of 60 healthy women 20–29 years of age, Shiraishi and Bezerra (2016) demonstrated significant improvements in core endurance in the intervention group following a 6-week (thrice weekly) yoga program. Our findings also corroborate with those of Cowen and Adams (2005), who observed a significant increase (improvements of 52–57% depending on the style of yoga) in muscle endurance in a group of university students following 6-weeks of yoga practice. In a sample of 30 university students, Beazley et al. (2017) found significant improvements (*p*<0.0001) in core muscle engagement during yoga practice. Given that there is an 80% lifetime prevalence of back injury within the nursing profession (Edlich et al., 2004), and there is a strong correlation between increased core endurance and back injury prevention (Rickman et al., 2012; Silfies et al., 2015; Smith et al., 2008), yoga interventions may be particularly helpful with preventing back injury in future nurses.

This study demonstrates that undergraduate nursing students are reporting symptoms of poor mental health and physical health. Our findings revealed high rates of baseline mental health problems based on high depression, anxiety, and stress scores in this small sample of future nurses. In our study, one in five nursing students reported symptoms of severe depression, and one in four reported symptoms of severe anxiety or stress (Antony et al., 1998; Brown et al., 1997; Crawford & Henry, 2003; Lovibond & Lovibond, 1995). These findings are consistent with a 2019 survey of 67,972 students attending Canadian post-secondary institutions (American College Health Association, 2019) which showed that 51.6% felt so depressed it was difficult to function, 68.9% felt overwhelming anxiety and 60.9% reported above average stress levels over the previous 12 months. Additionally, a study of nursing students relative to the general student body (*N*=2104) at a major American university showed that nursing students reported significantly higher stress rates than the general student body (*p*=0.0001). Future research should focus on the genesis of these differences, and ways to improve them.

The majority of students in our study demonstrated below average core endurance (86.8%) at baseline. In a study of 98 nursing students Irazusta et al. (2006) found that up to 50% were sedentary. Participants in our study showed significant improvements in core endurance (baseline mean 68.19 s to post intervention mean of 108.51 s; *p*<0.001). These findings are particularly relevant given the strong relationship between core endurance, alleviation of low back pain, and musculoskeletal injury prevention (Akuthota et al., 2008).Our study addresses methodological weaknesses identified in previous studies of this type (Ciezar-Andersen et al., 2021). A key limitation of previous studies has been lack of reproducibility, addressed here through the use of both the Essential Properties of Yoga Questionnaire (EPYQ) and TIDieR Checklist tools in the design of the intervention (Ciezar-Andersen et al., 2021). Use of accessible pre-recorded yoga classes to conduct the intervention ensured that all intervention details are available and reproducible in accordance with the TIDieR Checklist (Hoffmann et al., 2016). Additionally, well-validated tools (DASS-42, SCS, MCET) were used in the present study to measure symptoms of mental and physical health, which was an identified shortcoming of previous studies. While this study has also demonstrated the potential of delivering a yoga intervention via a flexible online platform across multiple sites, future studies could focus on optimizing acceptability to minimize attrition.

### 4.1 Limitations

The results of our study should be interpreted with caution. A key limitation of this study was the self-selection of participants, and the pre-post study design. Recruitment required multiple strategies to attain the sample and retention was poor (attrition was 44% of those who started the study). The majority of those who withdrew from the study cited mounting demands on their time as the main reason. The participants were also a homogenous group (predominantly female aged in their mid-twenties). Both of these factors render limited generalizability of the study findings. Finally, we did not follow-up participants beyond the 6-week conclusion. It would be worth investigating more long-term benefits of a yoga intervention.

## 5. Conclusion

Research exploring interventions to improve mental and physical health of undergraduate nursing students is limited. Our study demonstrated that an online yoga intervention has the potential to significantly improve participant mental health (i.e., symptoms of depression, stress, anxiety, and self-compassion) and physical health (i.e., core endurance). New nursing graduates enter a highly psychologically and physically demanding profession plagued by high burnout rates (Laschinger et al., 2010; Rudman & Gustavsson, 2011, 2012; Sveinsdóttir et al., 2021; Valero-Chillerón et al., 2019). Given the success of this intervention, post-secondary institutions might wish to consider implementing similar low-cost (cost of optional yoga mat) flexible interventions with high-risk student cohorts. Large scale RCT studies are recommended to collaborate findings of this pre-post design.
